# Mental health and well-being of university staff during the coronavirus disease 2019 levels 4 and 5 lockdown in an Eastern Cape university, South Africa

**DOI:** 10.4102/sajpsychiatry.v27i0.1589

**Published:** 2021-03-08

**Authors:** Rudolph L. van Niekerk, Maria M. van Gent

**Affiliations:** 1 Department of Human Movement Sciences, Faculty of Health Sciences, Fort Hare University, Alice, South Africa

**Keywords:** COVID-19, pandemic, mental health, mental well-being, university, staff members, risk, academic

## Abstract

**Background:**

The mental health of university staff members is often neglected and might have been exacerbated during the coronavirus disease 2019 (COVID-19) pandemic.

**Aim:**

The aim of this study was to determine the mental health and well-being of staff members in an Eastern Cape university just after levels 4 and 5 lockdowns (01 June 2020) in South Africa.

**Setting:**

The university was closed during lockdown and staff members had to work from home, trying to save the 2020 academic year.

**Methods:**

A cross-sectional exploratory survey of a sample of 280 staff members (response rate = 27.75%), with a mean age of 48.84 ± 10.17 years, completed the Kessler Psychological Distress Scale (K10) and Mental Health Continuum – Short Form (MHC-SF).

**Results:**

A number (27.6%) of staff members reported psychological distress, whilst the majority (60%) was flourishing during lockdown. Socio-economic collapse, contracting the virus and the completion of the academic year were their biggest worries. Whilst a strong negative correlation between psychological distress and mental well-being (MWB) was observed (*r* = −0.595), age had an inverse correlation with psychological distress (*r* = −0.130) and a positive correlation with MWB (*r* = 0.153). Female staff members, staff members with comorbidities and workers in the administration and service sections were significantly more likely to report psychological distress. The mental health of female staff members and members with comorbidities were almost two times more at risk for psychological distress.

**Conclusion:**

The mental health and well-being of some university staff members were at an increased risk during lockdown.

## Introduction

The coronavirus disease 2019 (COVID-19) pandemic because of the worldwide outbreak of the severe acute respiratory syndrome coronavirus 2 (SARS-CoV-2)^1^ had an unprecedented effect on the daily lives of people.^2^ A national lockdown restricting the operation of all non-essential businesses and services, including the closure of all South African universities, followed.^3^ This disruption put the mental health of university staff members at risk.^4^ Levels 4 and 5 lockdown served as a drastic intervention to contain the spread of the virus and saved lives and resulted in the restriction of university staff members to their homes. It posed a direct threat to the successful progression and completion of the 2020 academic year for universities^4^ and its impact on the mental health of staff and students is of concern. The mental health of South African university personnel has been largely neglected and was probably exacerbated by the COVID-19 pandemic.^5^

Statistics revealed a global infection of 12 322 393 cases and 556 335 reported deaths (at the time of data collection) because of the coronavirus, including an infection of 250 687 people and 3860 reported deaths in South Africa.^6^ The Eastern Cape province was regarded as a ‘hotspot’ for the spread of the disease, reporting the third largest number of infected cases (44 432) following Gauteng province (81 546) and the Western Cape province (74 815) in South Africa.^7^

The United Nations^2^ emphasised that the effect of the COVID-19 pandemic on the mental and psychological well-being (PWB) of people cannot be underestimated and released guidelines^8^ to address the elevated rates of stress and anxiety observed. Fear, worry and anxiety are typical reactions to perceived threats like a pandemic, and people should take care of their mental health and well-being during the outbreak of the virus. Major mental health implications should be expected, as the current burden of mental health disorders, past and present trauma, poverty and the unexpected displacement of people in South Africa pose particular challenges in this regard.^9^

Anxiety and worry on a personal and institutional level are evident.^10^ As mental health took a backseat to physical health in the global reaction to the COVID-19 pandemic (isolation through lockdown), the impact on people’s mental health is neglected. A comparative study of the mental health of American adults during the pandemic with results prior to the pandemic from 2018 found that participants in the pandemic were eight times more likely to screen positive for severe mental health, with 70% of the participants (compared with 22% in 2018) meeting the criteria for serious to moderate mental illness.^11,12^ The South African Depression and Anxiety Group^13^ survey reported that 65% of participants felt stressed and severely stressed about the spread of coronavirus, finances, relationship problems, job security, grief, gender-based violence and trauma.

Increases in depression and anxiety amongst staff members in higher education are expected globally.^14^ Reports prior to the pandemic showed an escalation in the poor mental health of university staff members in the United Kingdom^15^ and South Africa.^16^ Factors such as work-related stress, high workloads, often unattainable performance evaluation processes, the competitive environment in academia and contract employment,^15^ the sudden demand to adjust to online teaching, ambiguous boundaries between work and home and social disconnection from students,^4^ additional administrative duties and limited organisational support^16^ are highlighted. Sahu^17^ described the potential impact of the pandemic on the mental health of university staff members as a ‘sense of uncertainty and anxiety about what is going to happen’.

Evidence of the impact on the mental health of university staff members seemed to indicate a shift from work-related stress to anxiety as the main concern amongst staff members.^18^ Staff members’ ability and skills to deliver teaching in the current context (online) is a primary contributor to their heightened anxiety levels.

University employees experienced the pandemic in three phases^18^: phase 1: uncertainty and instability, demanding an adjustment to the work context; phase 2: fatigue, characterised by new realities and new fears; and phase 3: re-opening, generating new feelings of uncertainty amongst staff members. Studies showed an increase in the mental health problems of American^19^ and Spanish^20^ university staff members, specifically for anxiety, depression and stress.

The two-continua model of mental health and well-being constitutes both an illness (mental health) and a health perspective (wellness).^21^ Whilst mental health is defined as ‘a dynamic state of internal equilibrium, which enables individuals to use their abilities in harmony with universal values of society’,^22^ mental well-being (MWB) is defined as:

[*A*] state of well-being in which the individual realises his or her own abilities, can cope with the normal stresses of life, can work productively and fruitfully and is able to make a contribution to his or her community.^23^

For mental health, a person is screened for and diagnosed in order to identify mental illnesses and disorders (e.g. Diagnostic and Statistical Manual [DSM-5]^24^) and for MWB, a combination of a person’s hedonic (emotional) and eudaimonic (social and psychological)^21^ well-being is indicative of either flourishing, moderate mental health or languishing individuals.^25^ Thus, the aim of this study was to determine the mental health and well-being of university staff members at an Eastern Cape university during levels 4 and 5 lockdown.

## Methodology

A cross-sectional explorative study was undertaken to determine the mental health and well-being of staff members at an Eastern Cape university during the COVID-19 pandemic levels 4 and 5 lockdown. The university is one of the four universities in the Eastern Cape and comprised 371 academic and research staff, and 597 administrative/support services staff members.^26^ The academic, administrative and service staff members (*n* = 1009) spread over three campuses were invited via their university email address to participate in the study. Temporary (time on task) employers, outsourced services staff (e.g. cleaners, gardeners and security guards) and visiting academics at the university were excluded from the study.

A sample size of 278 participants (https://www.surveysystem.com/sscalc.htm) was expected to achieve statistical power at a 95% confidence interval and 0.05 significance level. A purposive sample of 280 staff members (response rate = 27.75%) completed the questionnaire (in English) and submitted their responses electronically. Participants completed a questionnaire on an electronic platform (Survey Monkey©) via a link provided to staff members on either their cell phones or computers. The questionnaire comprised a biographical section and two validated psychometric tests. Apart from biographical questions (gender, age, etc.), the authors decided to include questions on the occupational (work roles, rank and years working) and environmental context (living environment and people) of the participants during lockdown as it would describe the working environment of the participants during lockdown. As the premise of the two-continua model holds that mental health and mental illness are two distinct, but related constructs,^21^ the authors decided to explore participants’ mental from both the illness perspective through the Kessler Psychological Distress Scale (K10)^28^ and the health perspective through the Mental Health Continuum – Short Form (MHC-SF).^25^

These two tests assessed participants’ mental health from both an illness and health perspective as conceptualised in the two-continua model:

- The Kessler Psychological Distress Scale (Kessler et al.^27^) is a 10-item test, screening for mental health concerns and indicating non-specific psychological distress with validated cut-off points for serious DSM-V mental illnesses. It elicits responses on a five-point Likert scale and is used globally in the World Health Organization (WHO) World Mental Health Initiative as a screening tool for mental health. Two studies^28,29^ reported excellent reliability and validity for the instrument in specific populations in South Africa. However, the K10 showed only a moderate discriminant ability to detect disorders in the general South African population^30^ and thus caution in the interpretation of the results is suggested because of a lack of clinical norms for culturally diverse groups.^31^
- The Mental Health Continuum – Short Form^25^ is a 14-item questionnaire with answers on a six-point Likert scale. The test provides information on three subscales, measuring PWB, emotional well-being (EWB), social well-being (SWB) and an overall global well-being score. The test has been validated for Setswana-speaking South Africans^32^ and rendered acceptable to high reliability on all subscales. As most of the participants in this study are isiXhosa-speaking people, the reliability of both instruments will be reported.

Data were analysed in Statistical Package for Social Sciences (SPSS) version 25.^33^ Participants with incomplete data on items of both the tests were excluded from the analysis. Descriptive statistics were calculated for each domain of the mental health (K10) and well-being (MHC-SF) scores. Scales and subscales were calculated and interpreted according to the manuals and guidelines for the different tests. Categorisation was carried out according to the norms and norm scores of the tests. Reliability of the scales and subscales was determined with Cronbach’s alpha as a measure of the internal consistency. Alpha was set at 0.05, with a confidence interval of 95% for all statistical analyses. Correlation coefficients were calculated and group comparisons were made with independent sample *t-*tests and one-way analysis of variance. Logistical regression was carried out to determine the factors that predict and contribute significantly to the mental health risk of the participants.

### Ethical considerations

Ethical approval was obtained from the Health Research Ethics Committee at the University of Fort Hare (REC-100118-054-Ref# 2020=05=002=RLvanNiekerk) and permission was obtained from the Registrar of the university to conduct the study. Participants provided informed consent before participation in the study. Confidentiality and anonymity were ensured for personal information. Participants were given the option to leave contact details for referral to psychological services where necessary. Participation was voluntary and anyone could withdraw from the study at any point if they wished so.

## Results

A sample of 280 staff members of the university (mean age of 48.84 ± 10.17 years) and a gender distribution of 121 (43.7%) men and 156 (56.3%) women participated in the study. The roles included 148 (53.8%) academic and research staff, 95 (34.5%) administrative staff and 32 (11.4%) service staff members. The role distribution of the staff members is presented in Table 1.

**TABLE 1:** Distribution of participants according to their roles in the university.

Academic and research role			Administrative role			Service role		
Position	*n*	%	Position	*n*	%	Position	*n*	%
Professor	17	11.5	Senior manager	8	8.5	Infrastructure services	7	20
Assistant professor	21	14.2	Mid-level manager	5	5.3	Student services	12	34.3
Senior lecturer	30	20.3	Manager	16	17	Staff services	6	17.1
Lecturer	66	44.6	Head of department	1	1.1	Academic services	4	11.4
Junior lecturer	14	9.5	Section head	6	6.4	IT services	3	8.6
-	-	-	Administrator	58	61.7	Financial and legal services	3	8.6

IT, information technology.

Most of the academic and research staff were from the Social Sciences and Humanities Faculty (*n* = 53, 28.2%), followed by the Faculties of Science and Agriculture (*n* = 37, 19.7%), Health Sciences (*n* = 31, 16.5%), Management and Commerce (*n* = 31, 16.5%), Education (*n* = 26, 12.8%) and Law (*n* = 12, 6.4%).

Of the participants, 184 (66.2%) were from an urban and 94 (33.8%) were from a rural area in the Eastern Cape province. The home environment (Table 2) during lockdown for most of the participants was a brick house with a yard (*n* = 220, 79.9%) and most reported more than four people (*n* = 61, 22%) with them in the house during lockdown. Only 26 (9.4%) of the participants lived alone.

**TABLE 2:** Home environment and the number of people in home during lockdown.

Home environment	*n*	%	Number of people in home during lockdown	*n*	%
Brick house with a yard	220	78.9	Alone	26	9.4
Brick house with no yard	13	4.7	One person	40	14.4
Flat	37	13.3	Two people	41	14.8
One room apartment	5	1.8	Three people	57	20.6
Informal house	1	0.4	Four people	52	18.8
Farm	3	1.1	More than four people	61	22.0

Results for the mental health and well-being of the staff members are presented in Table 3. All scales had high reliability, with Cronbach’s alpha between 0.847 and 0.907. Although the results are interpreted with caution as alluded to earlier, the high reliability scores provide some support for the usefulness of the K-10 as a screening tool and the MHC-SF as an indication of the MWB of staff members who participated in the study. Mental health (K-10) categories indicated that 195 (72.5%) of the staff members were mentally well and 27.6% (*n* = 74) had mild to severe levels of psychological distress. This result could not be compared with either a baseline measure or other studies to determine the impact of the COVID-19 pandemic on the mental health of staff members. The MWB (MHC-SF) scores indicated that most staff members were flourishing (*n* = 151, 60.6%), whilst 39.3% (*n* = 98) had moderate levels of well-being or were languishing. Thus, between 27.6% and 39.3% of staff members might be at risk of developing mental health problems. On the subscales of the MWB scale (MHC-SF), 58.9% and 68.2% participants reported high emotional and PWB, respectively, whilst only 36.4% reported high SWB. It is evident that most of the participants’ SWB was negatively impacted.

**TABLE 3:** Descriptive results and reliability of the mental health and wellness scores.

Variable	Mean	SD	Reliability	*n*	%
**Scale**					
Psychological distress (K-10)	17.74	7.15	0.907	-	-
Mental well-being (MHC-SF)	46.93	13.45	0.929	-	-
Emotional well-being (EWB)	10.55	3.25	0.888	-	-
Social well-being (SWB)	14.15	5.96	0.847	-	-
Psychological well-being (PWB)	22.21	6.11	0.907	-	-
**Mental health risk categories**					
Well	-	-	-	195	72.5
Mild psychological distress	-	-	-	26	9.7
Moderate psychological distress	-	-	-	23	8.6
Severe psychological distress	-	-	-	25	9.3
**Mental well-being categories**					
Flourishing	-	-	-	151	60.6
Moderate	-	-	-	81	32.5
Languishing	-	-	-	17	6.8

SD, standard deviation.

The top five biggest worries for participants were being infected by the coronavirus (49, 18.9%), not completing the academic year (32, 12.4%), uncontrollable spread of the virus (24, 9.3%), health and safety (21, 8.1%) and financial difficulty (19, 7.3%).

Previous diagnosis of mental disorders (e.g. depression, anxiety and dependency) and comorbid factors (e.g. diabetes, high blood pressure, high cholesterol and asthma) were also assessed. The results indicated that 25.7% of staff members had either a previous diagnosis of a mental disorder or a comorbid factor for coronavirus infection.

Correlations between variables, presented in Table 4, showed significant moderate to strong negative correlations between psychological distress and all the subscales of MWB, suggesting that an increase in mental health risk is associated with a decrease in MWB. A significant, but weak negative correlation was observed between age and psychological distress (*r* = −0.130, *p* = 0.033) and a significant, but weak positive correlation was found between age and MWB (*r* = 0.153, *p* = 0.011). Although very weak, the relationship could suggest that older staff members might be less at risk of psychological distress and more resilient to flourish.

**TABLE 4:** Correlations amongst age, mental health and mental well-being.

Variable	Statistic	Age	PD	MWB	EWB	SWB	PWB
Age	Correlation	1	-	-	-	-	-
	Significance	-	-	-	-	-	-
PD	Correlation	−0.130*	-	-	-	-	-
	Significance	0.033	-	-	-	-	-
MWB	Correlation	−0.153*	−0.595**	-	-	-	-
	Significance	0.011	0.000	-	-	-	-
EWB	Correlation	0.082	−0.632**	0.822**	-		-
	Significance	0.178	0.000	0.000	-	-	-
SWB	Correlation	0.169**	−0.471 **	0.891**	0.647**	-	-
	Significance	0.005	0.000	0.000	0.000	-	-
PWB	Correlation	0.129*	−0.515**	0.894**	0.645**	0.641**	-
	Significance	0.033	0.000	0.000	0.000	0.000	-

PD, psychological distress; MWB, mental well-being; EWB, emotional well-being; SWB, social well-being; PSW, psychological well-being.

* , Correlation is significant at the 0.05 level (2-tailed).

** , Correlation is significant at the 0.01 level (2-tailed).

Group comparisons showed no significant differences for the mental health and well-being scales between urban and rural communities (*p* > 0.05), between the different academic positions (*p* > 0.05), between different faculties (*p* > 0.05) and between their home environment and the number of people in the home during lockdown (*p* > 0.05). However, significant differences were observed between gender groups, staff roles and comorbidity as presented in Table 5. Female staff members, administrative and service personnel and participants with comorbidities had significant higher levels of psychological distress and lower levels of MWB than their counterparts.

**TABLE 5:** Group comparisons for gender groups, staff roles and comorbidity groups on psychological distress and mental well-being.

Variable	Group	*N*	Mean	SD	Sign.
**Gender**					
Psychological distress	Male	118	15.89	5.32	0.000
	Female	148	19.12	8.05	-
Mental well-being	Male	119	48.81	12.00	0.047
	Female	153	45.53	14.43	-
Emotional well-being	Male	119	11.15	2.67	0.008
	Female	153	10.09	3.59	-
Social well-being	Male	119	14.97	5.72	0.050
	Female	153	13.53	6.14	-
Psychological well-being	Male	119	22.68	5.42	0.293
	Female	153	21.90	6.60	-
**Roles of staff members**					
Emotional well-being	Academic	147	10.99	2.920	0.034
	Administrative	92	9.98	3.656	-
	Services	31	9.87	3.324	-
**Comorbidity**					
Psychological distress	Yes	70	19.21	7.306	0.045
	No	199	17.22	7.042	-
Mental well-being	Yes	72	43.79	15.150	0.021
	No	203	48.04	12.656	-
Emotional well-being	Yes	72	9.80	3.539	0.022
	No	203	10.82	3.109	-
Social well-being	Yes	72	13.23	6.260	0.128
	No	203	14.48	5.843	-
Psychological well-being	Yes	72	20.75	7.014	0.017
	No	203	22.73	5.687	-

SD, standard deviation; Sign., significance.

Finally, a direct logistic regression was performed to assess the impact of a number of factors on the likelihood that participants present with a mental health risk. The model contained four independent variables (gender, comorbidity, age and MWB). The full model containing all predictors was statistically significant, χ^2^ (4, *N* = 265) = 61.85, *p* = 0.000, indicating that the model could distinguish between respondents who did and who did not present with a mental health risk. The model as a whole explained between 20.8% (Cox and Snell R-squared) and 30.1% (Nagelkerke R-squared) of the variance in mental health risk and correctly classified 77.7% of cases. As shown in Table 6, only two of the independent variables made a unique statistically significant contribution to the model (gender and MWB). The strongest predictor of reporting a mental health risk was gender, recording an odds ratio of 1.96, which indicated that women were almost two times more likely to report a mental health risk than male staff members controlling for all other factors in the model.

**TABLE 6:** Factors contributing to the prediction of psychological distress.

Variable	*B*	SE	Wald	df	Sig.	Odds ratio	95% CI for odds ratio	
							Lower	Upper
Gender†	0.677	0.326	4.302	1	0.038	1.967	1.038	3.728
Comorbidity†	0.157	0.366	0.183	1	0.669	1.170	0.571	2.398
Age	−0.018	0.016	1.231	1	0.267	0.982	0.951	1.014
Mental well-being	−0.078	0.013	36.557	1	0.000	0.925	0.901	0.948
Constant	2.887	0.879	10.786	1	0.001	17.930	-	-

SE, standard error; CI, confidence interval; df, degrees of freedom; Sig., significance.

† , Variable(s) entered on step 1: gender, comorbidity, age and mental well-being.

## Discussion

At the time of data collection for this study, university staff members had been in lockdown (levels 4 and level 5) for almost 2 months working from home. The challenge at the time was to assist students and staff members to make a transition from face-to-face teaching to online teaching in a very short time.^4^ The pressure to make this transition and complete the 2020 academic year successfully had implications for academic, administrative and service personnel at the university. This study explored the mental health and MWB of staff members during that time.

Most of the staff members were in an environment during lockdown in homes that had enough space and companionship. This could be facilitative to their MWB, as psychological distress is usually associated with cramped living spaces and loneliness during lockdown.^34^ Whilst research on the size of living spaces found a weak relationship between the size of a home and well-being,^35^ the management of social space seemed more important than physical space when working from home, with smaller living spaces putting more strain on couples and families.^36^ Only a small proportion of staff members were alone during lockdown. However, research^37,38^ on loneliness during lockdown is considered a critical public health concern affecting mental health negatively.

Almost one-third of staff members reported non-specific mild-to-severe psychological distress after level 4 lockdown, who could be at risk of developing mental health problems. These results are fairly similar to those reported on depression, anxiety and stress of students and staff members at a Spanish university during COVID-19 lockdown.^20^ In this regard, female staff members were twice more likely to be at risk than male staff members. Although Lee^39^ found no gender differences for anxiety during lockdown, other research during the COVID-19 pandemic indicated a higher mental health risk amongst women.^40^

Administrative and service staff members had higher levels of psychological distress than academic staff members. On the contrary, administrative staff at a Spanish university reported lower anxiety scores, but higher depression scores than academic staff during lockdown.^20^ Staff members with comorbidities for infection with the coronavirus are of concern as their psychological distress is significantly higher than for staff members without comorbidities. Research^41^ found that hypertension, diabetes, chronic obstructive pulmonary disease (COPD), cardiovascular disease and cerebrovascular disease are major risk factors for patients with COVID-19. In addition, reports indicated that mental health disorder comorbidities to COVID-19 will make treatment more challenging and potentially less effective.^42^

The MWB of the staff members indicated that most (three in five) of the staff members were flourishing under lockdown conditions. However, the greatest impact was on the SWB of the participants. Lockdown implies forced isolation and restricted social contact with others, impacting negatively the SWB of university staff members, with three in five reporting moderate to low SWB. One study^43^ associated lockdown and its social isolation with negative psychological effects. The psychosocial well-being of both infected and uninfected people is significantly challenged by the COVID-19 pandemic,^44^ which is exacerbated by, amongst others, continuous lockdown time, fear of infection, frustration, financial loss and stigma.^43^ This coincides with the five biggest worries of staff members reported in this study, namely, fear of infection, not completing the academic year, family, unemployment and financial issues. These worries and fears contribute largely to the cost of well-being amongst the university staff members.

One of the strengths of this study was its quick access to participants via an online platform for research. It also contributes to a limitation to the study of participant bias, which was not controlled for. Another limitation associated with online questionnaires relates to access, excluding participants because of a lack of data or connectivity, which was problematic at the time of data collection. The high reliability and strong correlation between the psychometric tests (K-10 and MHC-SF) were strengths of this study, providing the authors with information that could be interpreted with confidence.

This study suggested that the risk of mental health and its impact on the MWB of staff members should not be underestimated. Studies found that university staff members showed a decrease in mental health^15^ and were under much pressure^16^ even before the COVID-19 pandemic, which is reinforced by exacerbating factors and new challenges to cope with online approaches to complete the academic year.^4^ It is recommended that efforts to minimise the impact of the COVID-19 pandemic on university staff and strengthen the resilience of staff members should be explored.^4^ Stress factors that reinforce the personal, environmental and work-related worries of staff members seem to be accentuated by findings of this study.

Guidelines on setting up a safe, healthy and functional workstation at home can be developed and communicated. Clear and timely communication of contingency plans for their personal safety and work conditions should reach staff members in an accessible way. Line managers should be alerted about the workload, skills development and ability of staff members to do their tasks successfully. As most of the work and meetings of staff members moved to an online platform, institutional and technical support for the capacitation of staff and students to get their work done should be implemented. Human resources and employee wellness programmes must be aimed at vulnerable groups, such as members who were alone in lockdown, female staff members and members with comorbidities, to inform them about functioning support systems and groups available. Information leaflets and educational material can be disseminated to create awareness about the symptoms of mental health problems amongst staff members and where to get help.

Strengthening the MWB and resilience of staff members could mitigate and lower the risk of mental health issues amongst university staff members. Future research could be focused on the development of models and programmes aimed at increasing the MWB of university staff members, both on a personal and institutional level.

## Conclusion

The mental health and well-being of university staff members was significantly impacted by the COVID-19 pandemic, leading to an increased risk of mental health issues such as depression, anxiety and stress and a decrease in MWB and a critical negative impact on the SWB of staff members.

Female staff members, staff members with comorbid factors for coronavirus infection and the impact on their mental health are of special concern. Exacerbating factors such as worries and fears of staff members should be addressed appropriately. Continuous efforts to build the resilience of staff members cannot be underestimated as mental wellness could mitigate the effect of the pandemic on the mental health of staff members.
